# Supplementary material for: A pcyt-1 Allelic Series Reveals In Vivo Consequences of Reduced Phosphatidylcholine Synthesis in C. elegans
Source: bioRxiv. 2026 Apr 25:2026.04.22.720214. Preprint. [Version 1] doi: 10.64898/2026.04.22.720214 (PMC13131500; doi:10.64898/2026.04.22.720214)
Supplement: 1 [file NIHPP2026.04.22.720214v1-supplement-1.pdf]

bioRxiv preprint doi: https://doi.org/10.64898/2026.04.22.720214; this version posted April 26, 2026. The copyright holder for this preprint (which was not certified by peer review) is the author/funder, who has granted bioRxiv a license to display the preprint in perpetuity. It is made available under aCC-BY 4.0 International license.

# Supplementary Figures

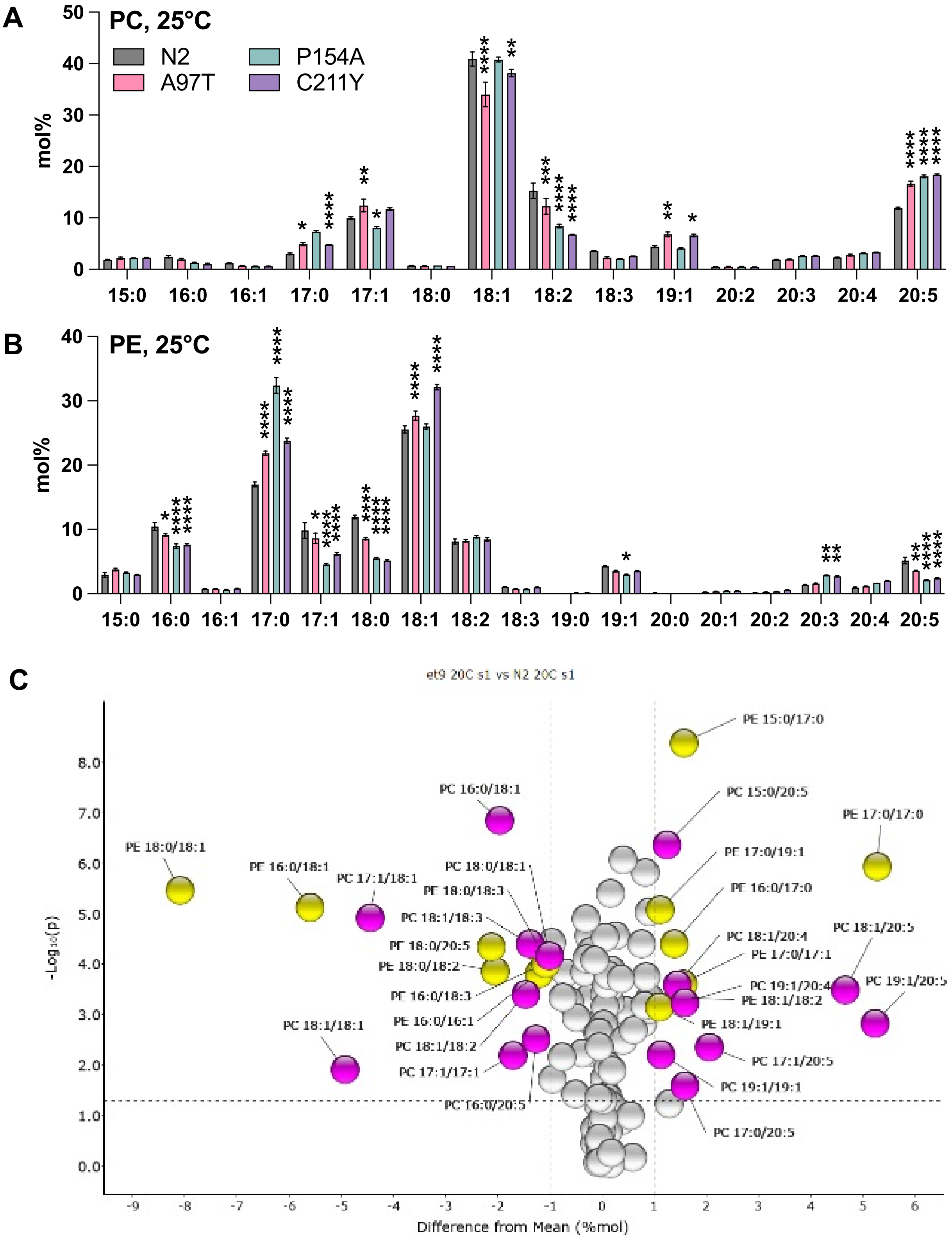

bioRxiv preprint doi: https://doi.org/10.64898/2026.04.22.720214; this version posted April 26, 2026. The copyright holder for this preprint (which was not certified by peer review) is the author/funder, who has granted bioRxiv a license to display the preprint in perpetuity. It is made available under aCC-BY 4.0 International license.

**Suppl. Fig. S1. Lipidomics reveals a hierarchy of *pcyt-1* allele 25°C. (A)** and **(B)** Show the fatty acid composition of phosphatidylcholines and phosphatidylethanolamines of L4 worms growth at 25°C and of the indicated genotypes; this data is included in the heat map from Fig. 6. For this analysis, the mol% fatty acid composition of phosphatidylcholines and phosphatidylethanolamines was determined using UPLC-MS/MS. For each fatty acid, the mean mol% among the samples was determined and set to 0 (zero) and the variance adjusted to 1, thus giving equal weight to all fatty acids. Note that the 15:0, 17:0 and 19:0 species likely consist mostly of mmBCFAs while the 17:1 and 19:1 species are likely mostly the dietary cyclopropanes 17:0 delta and 19:0 delta, respectively. **(C)** Volcano plot of PC and PE species in *pcyt-1(C211Y)* vs control N2 worms.

**Suppl Movies 1-4**

Z-stack of PCYT-1::AID::3XFLAG (WT for Movies 1 and 3; P154 variant for Movies 2 and 4) grown for 24 hours at either 20°C (Movies 1-2) or 25°C (Movies 3-4) post L1 stage.
